# The underestimated impact of excess body weight on colorectal cancer risk: Evidence from the UK Biobank cohort

**DOI:** 10.1038/s41416-023-02351-6

**Published:** 2023-07-13

**Authors:** Fatemeh Safizadeh, Marko Mandic, Dianne Pulte, Tobias Niedermaier, Michael Hoffmeister, Hermann Brenner

**Affiliations:** 1 Division of Clinical Epidemiology and Aging Research, German Cancer Research Center (DKFZ), Heidelberg, Germany; 2 Medical Faculty Heidelberg, Heidelberg University, Heidelberg, Germany; 3 Division of Preventive Oncology, German Cancer Research Center (DKFZ) and National Center for Tumor Diseases (NCT), Heidelberg, Germany; 4 German Cancer Consortium (DKTK), German Cancer Research Center (DKFZ), Heidelberg, Germany

**Keywords:** Colorectal cancer, Epidemiology

## Abstract

**Background:**

The association between excess weight and colorectal cancer (CRC) risk may have been underestimated due to potential weight loss during pre-clinical sojourn time of CRC. We aimed to investigate this association and the corresponding population attributable fraction (PAF), accounting for prediagnostic weight loss.

**Methods:**

Data from the UK Biobank prospective cohort were used. Multivariable adjusted hazard ratios (HR) and their 95% confidence intervals (CI) for various periods of follow-up and the corresponding PAF of excess weight were calculated.

**Results:**

During a median of 10.0 years of follow-up, of 453,049 participants, 4794 developed CRC. The excess weight–CRC association became substantially stronger with including increasing lengths of follow-up in the analyses and further excluding the initial years of follow-up. HRs (95% CIs) for overweight and obesity were 1.06 (0.97–1.16) and 1.14 (1.03–1.26) after 7 years of follow-up, 1.13 (1.05–1.21) and 1.23 (1.14–1.33) when including complete follow-up length, and 1.26 (1.12–1.43) and 1.42 (1.24–1.63) when excluding the initial 7 years of follow-up. The corresponding PAFs of excess weight were estimated as 6.8%, 11.3%, and 19.0%, respectively.

**Conclusions:**

Comprehensive consideration of the potential effect of prediagnostic weight loss discloses a much stronger impact of excess body weight on CRC risk than previously assumed.

## Background

Excess body weight, often represented as increased body mass index (BMI), is associated with higher risk of different cancers including colorectal cancer (CRC) [1, 2]. The incidence of CRC is growing especially in younger populations and formerly low-risk countries in which changes in life-style factors, including diet, e.g., higher meat consumption, sedentary life-style, and less physical activity resulting in excess body weight and unfavourable body fat distribution, play an important role [3]. The prevalence of overweight (BMI ≥ 25 to 30 kg/m^2^) and obesity (BMI ≥ 30 kg/m^2^) has increased rapidly over the last few decades [4].

The population attributable fraction (PAF) of overweight and obesity for CRC has been estimated between 5 and 11% in different populations [5–9]. Furthermore, recent systematic reviews and meta-analyses have reported approximately 18% and 30% increase in CRC risk for overweight and obesity, respectively, compared to normal weight [10, 11]. However, there are concerns that the impact of excess body weight may have been underestimated in epidemiological studies due to prediagnostic weight loss [12], which is common among CRC patients [13]. This particularly applies to case-control studies, in which BMI is commonly ascertained close to the time of diagnosis among cases. Nonetheless, prediagnostic weight loss may also affect risk estimates from cohort studies, where BMI is ascertained at baseline prior to diagnosis of CRC during follow-up. The mean sojourn time (average duration of pre-clinical phase) of CRC has been estimated 3 to 6 years [14–16], and a relevant proportion of cancers diagnosed in the early years of follow-up may have existed and led to weight loss already at recruitment.

We aimed to assess the potential role of prediagnostic weight loss during pre-clinical sojourn time on estimates of the excess weight–CRC risk association in a large prospective cohort with about half a million study participants, the UK Biobank.

## Methods

### Study design and study participants

The UK Biobank is a prospective cohort that has collected extensive data on socio-demographic, life-style, and health-related factors through a self-completed touch-screen questionnaire and a computer assisted interview from about half a million study participants across UK aged 40–69 years when recruited (2006–2010). The participants have also undergone physical and functional measurements and cancer, death, and primary care data are available through linkage to electronic health records as previously described elsewhere [17]. The UK Biobank has ethical approval from the North West Multi-centre Research Ethics Committee (MREC) as a Research Tissue Bank (RTB), approval renewed in 2021 (21/NW/0157) and all participants provided electronic signed informed consent. This analysis was restricted to men and women with no previous cancer diagnosis and complete information on BMI at recruitment.

### Assessment of exposure

BMI values were determined by dividing weight in kilograms by the square of height in metres. Weight was measured using the Tanita BC-418 MA body composition analyser and standing height using a Seca 202 height measure as part of the initial assessment visit [18]. BMI (kg/m^2^) was classified according to the World Health Organization (WHO) categories:  <18.5 (underweight), ≥18.5 to <25 (normal weight), ≥25 to 30 (overweight), ≥30 to <35 (obesity class I), ≥35 to <40 (obesity class II), and ≥40 (obesity class III) [19].

### Assessment of outcome

The coded data on cancer incidence (10th revision of the International Statistical Classification of Diseases (ICD-10)) are provided by the UK Biobank through linkage to national cancer registries. This analysis is based on cancer incidence follow-up to 31 July 2019 for England and Wales and 31 October 2015 for Scotland. CRC cases were defined as incident malignant neoplasms of the colon (C18), rectosigmoid junction (C19) and rectum (C20).

### Assessment of covariates

Data regarding socio-demographics, life-style, health, and medical history and medication use were collected at initial assessment visits. Age, ethnicity (white, Asian, black, mixed, and other), socio-economic status (Townsend deprivation index), educational qualifications (higher academic/professional, lower academic/vocational, or none), smoking status (never, former, current) alcohol consumption (never, at special occasions only, one to three times a month, once or twice a week, three or four times a week, daily, or almost daily), and level of physical activity (low, moderate, and high) according to international physical activity questionnaire (IPAQ) [20] were ascertained. Frequency and type of food intake collected via touch-screen questionnaire was used to determine fruit (pieces/day), vegetable (tablespoons/day), and red and processed meat intake, categorised into never, less than once a week, once a week, and two or more times a week.

Family history of CRC, history of bowel cancer screening (faecal occult blood test, colonoscopy/sigmoidoscopy), and regular use of non-steroidal anti-inflammatory drugs (NSAIDs) or aspirin were also identified.

### Statistical analysis

The baseline characteristics of the cohort are displayed with descriptive statistics. Median (interquartile range (IQR)) of BMI is reported by levels of covariates and compared by Kruskal–Wallis test.

Multivariable Cox proportional hazards models were used to evaluate the association between BMI and the risk of CRC. Follow-up time was defined as the time from cohort entry to the first CRC diagnosis, date of death, date lost to follow-up, or end of follow-up (31 July 2019 for England and Wales and 31 October 2015 for Scotland). Two models were fitted; the first model was adjusted for age (continuous) and sex and the second model was additionally adjusted for ethnicity, socio-economic deprivation (continuous), educational qualification, smoking status, alcohol consumption, physical activity, fruit (continuous), vegetable (continuous), red meat and processed meat consumption, family history of CRC, history of bowel cancer screening, and regular use of NSAIDs or aspirin. Deviations from the proportionality assumption were examined by Schoenfeld residuals plots for each covariate and no deviations were found. Missing covariate values were imputed using PROC MI and the analyses were performed combining the analyses of five imputed datasets using PROC MIANALYZE. The percentage of missing values was 20% for physical activity, and less than 2% for other covariates.

Hazard ratios (HRs) and corresponding 95% confidence intervals (CIs) were calculated to quantify the risk of CRC per each category of BMI compared to the normal weight as the reference group. The initial analysis used all of the above-mentioned WHO categories. In further analyses, combined categories <25 (normal weight), ≥25 to <30 (overweight), and ≥30 kg/m^2^ (obesity) were used due the small numbers of participants with BMI < 18.5 and >35 kg/m^2^.

We first carried out a standard cohort analysis using the full follow-up time available at the time of the analysis. Next, we repeated the analyses, starting with including only the first year of follow-up and then gradually increasing the maximum follow-up time (i.e., the censoring time) in steps of 1 year up to the maximum possible follow-up time of 13 years. The rationale was to gain more insight into the association between BMI and CRC risk in the early years of follow-up, when a substantial proportion of newly diagnosed CRC cases would be expected to have occurred among participants who already had pre-clinical CRC at the time of recruitment, and to see how length of follow-up might affect the derived overall estimates of the BMI-CRC association.

Subsequently, in order to progressively reduce potential bias from prediagnostic weight loss, we first excluded the first year of the follow-up time, which corresponds to a late entry analysis, and then extended this time to 2, 3, 4, 5, 6, 7, and finally first 8 years of follow-up. This was accomplished by excluding participants with follow-up time ≤1, ≤2, ≤3, ≤4, ≤5, ≤6, ≤7, and ≤8 years, respectively, and reducing follow-up time by 1, 2, 3, 4, 5, 6, 7, and 8 years, respectively, among the remaining participants.

We also calculated population attributable fractions (PAFs) that estimate the proportion of CRC cases in the study population that are statistically attributable to these conditions, using Levin’s formula [21]. Due to the predominant occurrence of CRC cases among older and male participants, who have higher prevalences of excess weight, we first calculated age- and sex-specific PAFs and derived the overall PAF as a weighted average of the age- and sex-specific PAFs, with weights equal to the age- and sex-specific numbers of CRC cases.

In addition, subgroup analyses by age group, sex, smoking status and history of bowel cancer screening, and subsite-specific analyses for colon cancer (C18.0–C18.9), proximal colon cancer (C18.0 and C18.2–C18.4), distal colon cancer (C18.5–C18.7), and rectal cancer (C19 and C20) were performed. For each subgroup and each subsite, HRs and PAFs were estimated for three types of analyses: (i) including the initial 7 years of follow-up only, (ii) including the entire follow-up, and (iii) excluding the initial 7 years of follow-up. Furthermore, we investigated the potential interaction between BMI (continuous) and each stratification variable by including the corresponding interaction term in the model. Differences regarding site-specific associations were evaluated by heterogeneity test for colon vs rectal cancer and proximal vs distal colon cancer. Both evaluations were conducted for the complete follow-up time.

All analyses were performed using SAS software version 9.4. Associations with two-sided *p*-values <0.05 were considered statistically significant.

## Results

**Fig. 1:**
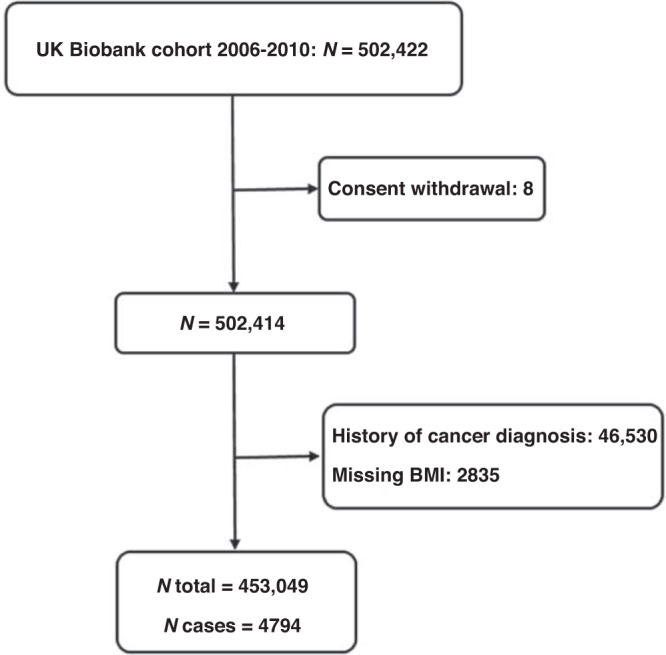
Study Flowchart. Flowchart showing selection of the study population.

Of 502,422 participants, 8 withdrew consent, 46,530 had cancer at or prior to recruitment and 2835 had missing values for BMI and were excluded from the analyses (Fig. 1). Therefore, 453,049 participants including 4794 CRC cases with median follow-up time of 10.0 years were included in the analyses.

In the total cohort, the median of BMI was 26.8 kg/m^2^ (IQR: 24.2–29.9). Baseline characteristics of the study population with regard to their measured BMI at baseline are shown in Table 1. The median BMI for men and women were 27.3 kg/m^2^ (IQR: 25.0–30.1) and 26.1 kg/m^2^ (IQR: 23.4–29.7), respectively, and participants who had older age, were male, black, had higher Townsend deprivation index, with no educational qualification, were former smokers, had higher frequency of alcohol consumption, and low physical activity had higher median BMI. Participants who consumed <2 and <3 portions of fruit and vegetables per day, or red or processed meat ≥2 times per week, reported history of bowel cancer screening, and family history of CRC also had higher median BMI.

**Table 1:** Baseline characteristics of the study participants according to body mass index.

Characteristics	*N* (%)	BMI (kg/m^2^) Median (IQR)	*p*-value*
Age at recruitment (years)			
<50	111,436 (24.6)	26.3 (23.6–29.5)	<0.0001
≥50 to <60	152,908 (33.8)	26.8 (24.1–30.1)	
≥60	188,705 (41.7)	27.0 (24.5–30.0)	
Sex			
Male	211,378 (46.7)	27.3 (25.0–30.1)	<0.0001
Female	241,671 (53.3)	26.1 (23.4–29.7)	
Ethnicity			
White	425,554 (94.4)	26.7 (24.1–29.9)	<0.0001
Mixed/other	6993 (1.6)	26.9 (24.2–30.3)	
Asian	10,790 (2.4)	26.2 (23.8–29.1)	
Black	7520 (1.7)	28.7 (25.8–32.3)	
Townsend deprivation index (quartiles)			
1 (most affluent)	113,118 (25.0)	26.4 (24.0–29.3)	<0.0001
2	113,108 (25.0)	26.6 (24.1–29.6)	
3	113,126 (25.0)	26.8 (24.2–30.0)	
4 (most deprived)	113,137 (25.0)	27.3 (24.4–30.9)	
Qualifications			
Higher academic/professional	221,771 (49.5)	26.2 (23.7–29.2)	<0.0001
Lower academic/vocational	150,180 (33.6)	27.1 (24.5–30.3)	
None	75,720 (16.9)	27.8 (25.1–31.1)	
Smoking status			
Never	248,730 (55.2)	26.5 (23.9–29.6)	<0.0001
Former	154,202 (34.2)	27.3 (24.7–30.4)	
Current	47,836 (10.6)	26.5 (23.8–29.6)	
Alcohol consumption			
Never	91,857 (20.3)	26.3 (23.9–29.0)	<0.0001
Special occasions only	104,922 (23.2)	26.4 (24.0–29.3)	
One to three times a month	116,980 (25.9)	26.8 (24.3–30.0)	
Once or twice a week	50,417 (11.2)	27.2 (24.4–30.8)	
Three or four times a week	51,647 (11.4)	27.6 (24.5–31.6)	
Daily or almost daily	36,202 (8.0)	27.4 (24.3–31.2)	
Physical activity (IPAQ groups)			
Low	68,317 (18.8)	27.8 (24.9–31.4)	<0.0001
Moderate	148,262 (40.7)	26.6 (24.1–29.7)	
High	147,381 (40.5)	26.3 (23.8–29.1)	
Fruit intake (pieces/day)			
<2	125,682 (27.8)	27.1 (24.5–30.2)	<0.0001
≥2 to <5	238,200 (52.8)	26.7 (24.1–29.9)	
≥5	87,561 (19.4)	26.3 (23.8–29.6)	
Vegetable intake (tablespoons/day)			
<3	81,859 (18.2)	27.0 (24.3–30.2)	<0.0001
≥3 to <6	227,303 (50.6)	26.6 (24.1–29.7)	
≥6	140,133 (31.2)	26.8 (24.2–30.0)	
Red meat intake			
Never	122,049 (27.0)	26.3 (23.6–29.6)	<0.0001
Less than once a week	278,626 (61.7)	26.8 (24.3–29.9)	
Once a week	48,177 (10.7)	27.5 (24.9–30.7)	
≥2 times a week	2849 (0.6)	28.1 (25.2–31.4)	
Processed meat intake			
Never	41,892 (9.3)	25.2 (22.8–28.3)	<0.0001
Less than once a week	136,526 (30.3)	26.4 (23.9–29.5)	
Once a week	131,645 (29.2)	26.9 (24.4–30.0)	
≥2 times a week	141,289 (31.3)	27.4 (24.8–30.6)	
History of bowel cancer screening			
Yes	135,197 (29.9)	26.9 (24.3–30.0)	<0.0001
No	316,902 (70.1)	26.7 (24.1–29.9)	
Family history of CRC			
Yes	48,663 (11.0)	26.9 (24.3–30.1)	<0.0001
No	395,035 (89.0)	26.7 (24.1–29.9)	
Regular use of NSAIDs/ aspirin			
Yes	139,040 (30.7)	27.7 (24.9–31.1)	<0.0001
No	313,993 (69.3)	26.4 (23.9–29.4)	

*p-values from Kruskal–Wallis test. The total number of participants might not add up to 453,049 for some covariates due to missing data. The percentages might not add up to 100 due to rounding.

*BMI* Body mass index, *IPAQ* International Physical Activity Questionnaire, *IQR* interquartile range, *SD* standard deviation, *CRC* colorectal cancer, *NSAIDs* non-steroidal anti-inflammatory drugs.

Table 2 shows the association with the risk of CRC for participants with underweight, overweight and obesity class I, II, and III compared to the normal weight participants, using the complete follow-up years included in the analyses. The results from model 1 and model 2 were very similar and therefore, only the results from the fully adjusted model are presented for further analyses. There was a 12% increase in CRC risk for overweight (HR: 1.12, 95% CI: 1.05–1.20), 21% for class I obesity (HR: 1.21, 95% CI: 1.11–1.31), 27% for class II obesity (HR: 1.27, 95% CI: 1.10–1.45), and 34% for class III obesity (HR: 1.34, 95% CI: 1.08–1.65). There was an inverse association between underweight and the risk of CRC but this association was not statistically significant (HR: 0.63, 95% CI: 0.35–1.14).

**Table 2:** Estimated hazard ratios and 95% CIs for incident CRC according to BMI categories with no exclusion of follow-up years.

Characteristic	*N* participants	Person-years	*N* cases	Hazard ratio (95% CI)	
				Model 1^a^	Model 2^b^
	453,049	4,537,473	4794		
BMI (kg/m^2^)					
<18.5 (underweight)	2318	22,686	11	0.63 (0.35–1.15)	0.63 (0.35–1.14)
≥18.5 to <25 (normal weight)	146,672	1,472,997	1264	Ref.	Ref.
≥25 to <30 (overweight)	192,936	1,933,856	2,196	1.13 (1.05–1.21)	1.12 (1.05–1.20)
≥30 to <35 (obesity class I)	79,584	794,580	963	1.22 (1.12–1.33)	1.21 (1.11–1.31)
≥35 to <40 (obesity class II)	22,711	225,940	264	1.28 (1.12–1.46)	1.27 (1.10–1.45)
≥40 (obesity class III)	8828	87,414	95	1.35 (1.10–1.66)	1.34 (1.08–1.65)
≥30 (overall obesity)	111,123	1,107,934	1323	1.24 (1.15–1.34)	1.22 (1.13–1.33)

*BMI* body mass index, *CI* confidence interval, *CRC* colorectal cancer, *Ref* Reference.

^a^Adjusted for age and sex.

^b^Adjusted for age, sex, ethnicity, socio-economic deprivation, education, smoking status, alcohol consumption, physical activity, fruit, vegetable, red meat and processed meat intake, history of bowel cancer screening, family history of CRC, and regular use of NSAIDs or aspirin.

Table 3 displays the HRs and PAFs for overweight and obesity compared to normal weight estimated with including various follow-up time windows after recruitment in the analysis. Within the initial four years of follow-up, null results with very small, statistically non-significant risk increases were estimated for overweight and obesity. Even after 8 years of follow-up, only a marginally significant 9% increase in risk was estimated for overweight participants. Only after inclusion of 10 or more years of follow-up, quite consistent estimates of significantly increased risk were obtained for both overweight (13–14% risk increase) and obesity (23–24% risk increase). However, even substantially higher estimates were obtained after excluding the initial years of follow-up, which might be affected by pre-clinical weight loss. For example, risk of CRC was estimated to be significantly increased by 16 and 31% beyond the fourth year of follow-up among overweight and participants with obesity, respectively. Risk estimates continued to increase with increasing length of excluded time window after recruitment until the 7th year of follow-up. The highest increase in CRC risk was seen for both overweight and obesity (increase by 26% and 42%, respectively) when 7 years of follow-up time were excluded. These risk increases are almost twice as high as the risk increases estimated when no follow-up years were excluded. Similarly, PAFs for overweight and obesity were estimated to be below 9% with follow-up times up to 8 years, 11.3% with the full period of follow-up (up to 13 years), but the estimate further increased to 19.0% after exclusion of the initial 7 years of follow-up.

**Table 3:**
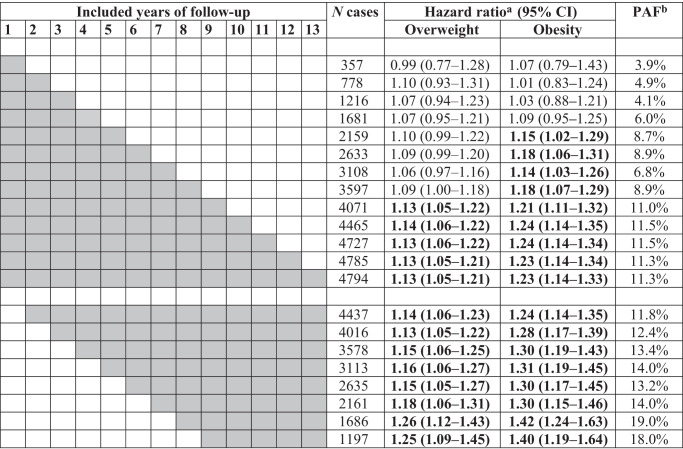
Hazard ratios (95% CIs) of CRC risk and population attributable fractions (PAFs) for overweight and obesity, obtained with inclusion of various follow-up time windows after recruitment in the analyses. Participants with BMI < 25 kg/m^2^ were the reference group in all analyses.

Gray fields show the follow-up years included in the analysis and significant HRs are shown in bold.

^a^Adjusted for age, sex, ethnicity, socio-economic deprivation, education, smoking status, alcohol consumption, physical activity, fruit, vegetable, red meat and processed meat intake, history of bowel cancer screening, family history of CRC, and regular use of NSAIDs or aspirin.

^b^Age- and sex-weighted average percentage of CRC cases that is estimated to be attributable to overweight and obesity.

Table 4 summarises results of the subgroup analyses, which show similar patterns as those observed in the main analyses. In all subgroups, the lowest increase in the risk of CRC or even no increase at all was observed for overweight and obesity during the initial 7 years of follow-up, and the increase in the CRC risk was higher when the initial 7 years of follow-up were excluded compared to when all the follow-up data were used. Overall, associations were stronger for older (≥50 years) than for younger (<50 years) participants, for men than for women, for former and current smokers than for never smokers, and for those with no history of bowel cancer screening. However, for some of the subgroup analyses, case numbers were rather low, which resulted in broad confidence intervals for some of the HRs. Point estimates of some HRs were also non-significantly lower than 1, which explains the apparent negative PAFs for some categories. Statistically significant interactions were observed for sex (*P*_interaction_ < 0.001) and history of bowel cancer screening (*P*_interaction_ = 0.04).

**Table 4:**
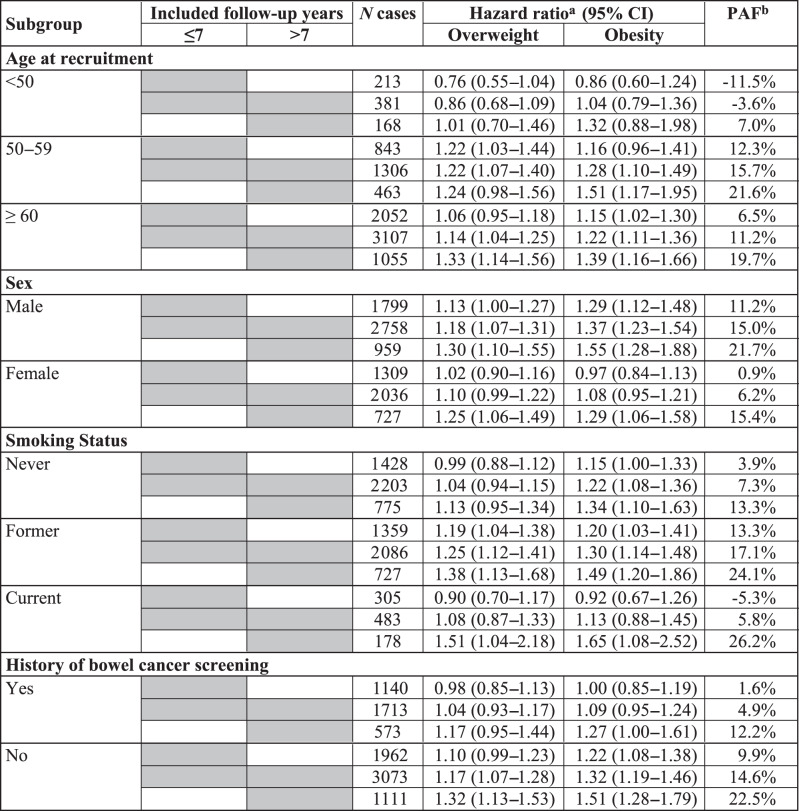
Subgroup-specific hazard ratios (95% CIs) of CRC risk and population attributable fractions (PAFs) for overweight and obesity, obtained with inclusion of various follow-up time windows after recruitment in the analyses. Participants with BMI < 25 kg/m^2^ were the reference group in all analyses.

Gray fields show the follow-up years included in the analysis.

^a^Adjusted for age, sex, ethnicity, socio-economic deprivation, education, smoking status, alcohol consumption, physical activity, fruit, vegetable, red meat and processed meat intake, history of bowel cancer screening, family history of CRC, and regular use of NSAIDs or aspirin.

^b^Age- and sex-weighted average percentage of CRC cases that is estimated to be attributable to overweight and obesity.

Table 5 shows the site-specific analyses for the association between overweight and obesity with the risk of colon, proximal colon, distal colon and rectal cancer separately. The association was stronger for colon cancer compared to rectal cancer in general, but heterogeneity by tumour site was not statistically significant (*P*_heterogeneity_ = 0.11), and quite similar for proximal and distal colon cancer (*P*_heterogeneity_ = 0.17). Furthermore, the risk increase was much stronger in the later years of follow-up than in the initial years of follow-up, with PAF estimates for both proximal and distal colon cancer reaching 24% in the later years of follow-up.

**Table 5:**
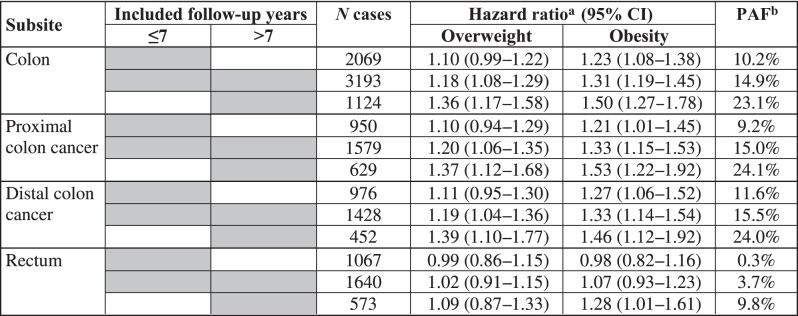
Site-specific hazard ratios (95% CIs) of colon and rectal cancer risk and population attributable fractions (PAFs) for overweight and obesity, obtained with inclusion of various follow-up time windows after recruitment in the analyses. Participants with BMI < 25 kg/m^2^ were the reference group in all analyses.

Gray fields show the follow-up years included in the analysis.

^a^Adjusted for age, sex, ethnicity, socio-economic deprivation, education, smoking status, alcohol consumption, physical activity, fruit, vegetable, red meat and processed meat intake, history of bowel cancer screening, family history of CRC, and regular use of NSAIDs or aspirin.

^b^Age- and sex-weighted average percentage of CRC cases that is estimated to be attributable to overweight and obesity.

## Discussion

In this large population-based cohort study, associations between excess weight and CRC risk were very weak or even absent during the initial years of follow-up, increased with increasing the length of follow-up, and were most pronounced in analyses based on long-term follow-up data in which the initial years of follow-up were excluded. These patterns are consistent with and support the hypothesis that the excess weight–CRC risk association may be substantially stronger than suggested by previous evidence from epidemiological studies with shorter follow-up or paying less attention to exclude or minimise bias due to prediagnostic weight loss.

Weight loss is a common symptom among CRC patients at different stages of the disease including the pre-clinical sojourn time [22–24], with estimated mean duration of 3 to 6 years [14–16]. Underlying mechanisms include increased catabolism and systemic inflammation caused by the tumour, which can lead to a negative energy balance and cancer cachexia [25, 26]. Such weight loss results in migration of patients with higher BMI to lower BMI categories, which in turn may result in weaker or even inverse associations of overweight and obesity with CRC risk.

Although cancer related weight loss has been recognised as a potential source of bias in epidemiological studies linking excess weight with CRC risk, in a recent umbrella review we provided evidence that many studies have not taken this potential bias into account in their analyses [27]. For instance, among the 21 cohort studies included in a recent systematic review and meta-analysis [11] evaluating the BMI-CRC incidence association, 15 studies (71%) did not implement any sort of exclusion of the first years of follow-up in their main analysis [28–42], only one study excluded more than the first year of follow-up [43], and, where conducted, sensitivity analyses mostly just excluded the initial 1 or 2 years of follow-up [29–32, 34, 36–38, 41]. In agreement with our findings, higher risk estimates were obtained in the sensitivity analyses in most cases. However, most studies were based on much smaller cohorts and none had provided a similarly comprehensive analysis of the potential impact of prediagnostic weight loss. Even though we aimed to minimise bias from prediagnostic weight loss, our results may still underestimate the true impact of excess weight. Even stronger effect estimates would be expected when life-time exposure to excess weight could be taken into account as recently demonstrated in a large study from Germany [44].

In a recent study from UK, the PAF of overweight and obesity was estimated as 13.3% for men and 5.6% for women [45]. These estimates, which were based on combining summary risk estimates from cohort studies and nationally representative survey data on prevalence of overweight and obesity in the UK, are comparable to our results (15.0% and 6.2%) with no exclusion of follow-up years, which according to our findings, is likely an underestimation of the CRC burden attributable to overweight and obesity. In another study, PAF estimates reported by sex and cancer site in 30 European countries [5], were lower for all countries compared to our results from UK, which may reflect both lower previous risk estimates and lower prevalence of overweight and obesity in most European countries compared to the UK.

In line with the results from recent systematic reviews [2, 46, 47] and the World Cancer Research Fund report [48], we observed weaker associations of overweight and obesity with CRC incidence for women compared to men, regardless of the length of follow-up included in the analysis, and therefore, lower PAFs of overweight and obesity were calculated for women. The Women’s Health Initiative trial [49], the European Prospective Investigation into Cancer and Nutrition (EPIC) cohort [50], and other studies and reviews [51–53], have pointed out the potential role of hormone replacement therapy (HRT) in post-menopausal women as an effect modifier in the BMI-CRC association, and women receiving HRT have been reported to be at lower risk of CRC. Endogenous sex-hormones have also been suggested as an explanation for the difference seen between men and women regarding CRC incidence, however the evidence has remained inconsistent. The weaker association due to HRT use might also be of relevance in our study population in which 37.5% of women reported receiving HRT at baseline.

A major strength of our study is use of the very large and comprehensive database of the UK Biobank with long-term follow-up and considerable numbers of participants and CRC cases, which allowed us to adjust for a broad range of covariates and to estimate risks at high levels of precision, even in the analyses restricted to shorter time windows of follow-up, and in subgroup and site-specific analyses. Most importantly, the BMI variable provided by the UK Biobank is based on highly standardised measurements rather than self-reported weight and height and is therefore not affected by reporting bias.

Our study also has limitations. First, due to the observational nature of the study, residual confounding may still exist despite adjustment for a large set of covariates. Second, despite the overall large number of participants and CRC cases, case number limitations still made reasonably precise risk estimates for underweight participants and obesity subclasses infeasible, which therefore had to be combined in most of our analyses. Third, our analyses exclusively focused on BMI as the most commonly used measure of excess weight in epidemiological studies. Central obesity measures such as waist circumference and waist-to-hip ratio (WHR) may be even stronger and more robust predictors of incident CRC than BMI [54–56] and should be addressed in future research. Fourth, the UK Biobank population mostly consists of white European participants and therefore, the results might not be generalisable to other populations.

Our analyses, based on the very large UK Biobank cohort, provide evidence that excess weight may account for a substantially larger share of the CRC burden than previously suggested. Future studies should pay more attention to avoid underestimation of the role of overweight and obesity due to prediagnostic weight loss by more rigorously considering timing of exposure measurement and taking cumulative life-time exposure rather than weight at a single point of time into account. Given the high and continuously increasing prevalence of overweight and obesity, this burden is expected to further increase and, along with demographic aging, will further accelerate the expected rise in numbers of CRC cases in many countries around the globe. Enhanced efforts to cope with the obesity epidemic will be crucial for more effective prevention of CRC as well as many other excess weight related cancers and diseases.

## Supplementary information

Strobe checklist

## Data Availability

Data was re-used with the permission of the UK Biobank. This work used data provided by patients and collected by the NHS as part of their care and support. The UK Biobank is an open-access resource and bona fide researchers can apply to use the UK Biobank dataset by registering and applying at https://www.ukbiobank.ac.uk/enableyourresearch/apply-for-access. Further information is available from the corresponding author upon request.

## Code availability

The code used to analyse the data and generate the results in this study is available from the UK Biobank resource for all researchers upon registration to access the UK Biobank data at https://www.ukbiobank.ac.uk/enableyourresearch/apply-for-access.
